# Predicting Complexity Perception of Real World Images

**DOI:** 10.1371/journal.pone.0157986

**Published:** 2016-06-23

**Authors:** Silvia Elena Corchs, Gianluigi Ciocca, Emanuela Bricolo, Francesca Gasparini

**Affiliations:** 1 Department of Informatics, Systems and Communication, University of Milano-Bicocca, Viale Sarca 336, 20126 Milano, Italy; 2 Department of Psychology, University of Milano-Bicocca, Via dell’ Innovazione 10, 20126 Milano, Italy; 3 Milan Center for Neuroscience, Milano, Italy; Ningbo University, CHINA

## Abstract

The aim of this work is to predict the complexity perception of real world images. We propose a new complexity measure where different image features, based on spatial, frequency and color properties are linearly combined. In order to find the optimal set of weighting coefficients we have applied a Particle Swarm Optimization. The optimal linear combination is the one that best fits the subjective data obtained in an experiment where observers evaluate the complexity of real world scenes on a web-based interface. To test the proposed complexity measure we have performed a second experiment on a different database of real world scenes, where the linear combination previously obtained is correlated with the new subjective data. Our complexity measure outperforms not only each single visual feature but also two visual clutter measures frequently used in the literature to predict image complexity. To analyze the usefulness of our proposal, we have also considered two different sets of stimuli composed of real texture images. Tuning the parameters of our measure for this kind of stimuli, we have obtained a linear combination that still outperforms the single measures. In conclusion our measure, properly tuned, can predict complexity perception of different kind of images.

## Introduction

The study of image complexity perception can be useful in many different domains. Within the human-computer interaction field, Forsythe et al. [1] proposed an automated system to predict perceived complexity and applied it in icon design and usability. Reinecke et al. [2] quantified visual complexity of website screenshots, formulating a model for the prediction of visual appeal in order to improve the user experience on the web. In addition it has been deemed useful in computer graphics, where a better understanding of visual complexity can aid the development of more advanced rendering algorithms [3] or image based 3D reconstruction [4]. Digital watermarking methods also can benefit from an estimation of image complexity as it has been related to the amount of information that can be hidden in image [5]. Nowadays it finds application in context-based image retrieval [6] where a model of joint complexity of images gives distances that can be used to estimate the degree of similarity between images. For instance in the research area of mobile visual search an hypothetical image complexity coefficient could aid within the image search process [7–9]. Within video quality research, the compression level and bandwidth allocation can be related to image complexity: low complexity stimuli can be compressed more easily and requires less bandwidth than an image with high complexity [10]. In the field of Automatic Target Recognizer, Peters and Strickland [11] proposed an image complexity metric to provide an a priori estimate of the difficulty of locating a target in an image. Moreover, object complexity is also considered as an object-level feature to predict saliency in the visual field [12, 13]. The authors introduced different attributes to predict saliency, and among them complexity. The image complexity concept is also used by neuroscientists interested in the mechanisms of recognition, learning and memory [14]. Recently, the complexity topic has been investigated across domains, using both visual and musical stimuli [15].

The different definitions of image complexity found in the literature depend on the specific task and the application domain. From a purely mathematical point of view, Kolmogorov [16] defined the complexity of an object as the length of the shortest program that can construct the object from basic elements, or description language. Snodgrass et al. [17] referred to the visual complexity as the amount of detail or intricacy in an image. Heaps and Hande [18] defined complexity as the degree of difficulty in providing a verbal description of an image. Image complexity is also related to aesthetics: Birkhoff [19] introduced the concept of aesthetic measure as the ratio between order and complexity.

As reviewed by [20], image complexity should be considered in relation to other factors like familiarity, novelty, and interest among others. Complexity is influenced not only by the spatial properties of the stimuli but also by the temporal dimension [21, 22]. Attempts to describe the image complexity using different mathematical models have been proposed, like for example fuzzy approaches [21, 23–25], independent component analysis [6] and information theory-based approaches [26].

Visual complexity can be also related to the concept of visual clutter. Rosenholtz et al. [27] proposed two measures of visual clutter: Feature Congestion (FC) and Subband Entropy (SE). They compared FC and SE performances with respect to the edge density used by Mack and Oliva [28].

Several researchers have conducted experiments to study the subjective perception of visual complexity. The state of the art studies differ in the kind of stimuli used during the experimental sessions and on the type of objective measures used to correlate the subjective scores.

During the experiments by Chikhman et al. [29], observers were asked to rank the complexity of two groups of stimuli: unfamiliar Chinese hieroglyphs and outline images of well-known common objects. To predict image complexity, they considered: spatial characteristics of the images, spatial-frequency characteristics, a combination of spatial and Fourier properties, and the size of the image encoded as a JPEG file. Oliva et al. [30] analyzed image complexity of indoor scenes. During their experimental session, participants performed a hierarchical grouping task in which they divide scenes into successive groups of decreasing complexity, describing the criteria they used at each stage. Their results demonstrated a multi-dimensional representation of visual complexity (quantity of objects, clutter, openness, symmetry, organization, variety of colors). Purchase et al. [31] conducted an experimental study using landscapes, domestic objects and city scenes as stimuli. The authors correlated their experimental results to a set of four computational metrics: colour, edges of objects, intensity variation, and file size. Cavalcante et al. [32] proposed a method to evaluate the complexity perceived in streetscapes images based on the statistics of local contrast and spatial frequency. Ciocca et al. [33] investigated the influence of color in the perception of image complexity, considering several visual features, that measure colors as well as other spatial properties. The perception of texture complexity has been studied by different authors [34–36]. Recently, Guo et al. [37] assessed the visual complexity of paintings, providing a machine learning scheme for investigating the relationship between human visual complexity perception and low-level image features.

Taking into account the multi-dimensional aspect of complexity, we propose a complexity measure based on a combination of several features related to spatial, frequency and color properties in order to predict complexity perception of real world images. We here consider two different kind of real world stimuli: Real Scenes, (RS) and real texture patches (TXT). The aim of our work is to propose a general purpose metric, that, tuned with respect to the kind of stimuli considered, can better correlate the subjective data with respect to single measures. We here propose a linear combination of visual features to predict image complexity perception where the weighting coefficients can reveal the role of each of them. Starting from a given set of stimuli, we apply a Particle Swarm Optimization (PSO) [38, 39] to find the weighting coefficients of the linear combination that best fits subjective data. We set up an experiment, where observers evaluated the complexity of real world scenes on a web-based interface and they were also asked to verbally describe the criteria that guided their evaluation.

Analyzing the most common criteria reported by the observers in the questionnaire, it could be possible to associate some of them with single image features and compare the frequency of these criteria to the weighting coefficients of the linear combination. To test our proposal we performed a second experiment on a new database of real world scenes, where the linear combination previously obtained is correlated with new subjective data. To verify the usefulness of our complexity measure to predict complexity of a different type of stimuli, we performed two more experiments on two different datasets of texture images. We have chosen texture images because they present a high range of complexity levels like real world scenes but with a different semantic content. We again apply PSO to find the new weighting coefficients of the linear combination proposed on the first of these two texture sets of images and we test the obtained measure on the other texture dataset. Up to our knowledge no supervised or unsupervised measure to evaluate complexity perception of real world images has been presented in the literature. Thus as a benchmark for evaluating our proposal, we consider two measures of visual clutter: FC and SE [27]. We have chosen these two measures as they have been frequently used in the literature, where they have shown correlation with image complexity perception, [3, 32, 40], even if they are not unsupervised measures and they were not specifically designed to predict image complexity.

## Materials and Methods

In this work we performed four experiments where the task is to evaluate image complexity. Each experiment is characterized by a different set of visual stimuli.

### Stimuli

The images used as stimuli are all of high quality, acquired with professional and semi-professional cameras.

In Experiment 1 we used 49 images depicting real world scenes (RS1 dataset) belonging to the personal photo collection of the authors (RSIVL [41]). For Experiment 2 we considered other 49 real world scenes (RS2 dataset). These images correspond to the reference high quality images of the LIVE [42–44] (29 images) and the IVL database [45, 46] (20 images). Images belonging to RS1 and RS2 have been chosen to sample different contents both in terms of low level features (frequencies, colors) and higher ones (face, buildings, close-up, outdoor, landscape). They include pictures of faces, people, animals, closeup shots, wide-angle shots, nature scenes, man-made objects, images with distinct foreground/background configurations, and images without any specific object of interest. Experiments 3 and 4 consider two different datasets of real texture images, that represent a kind of stimuli with contents significantly different from those represented within RS1 and RS2.

In Experiment 3, we consider 54 real texture images (TXT1 dataset), belonging to the VisTex data set [47]. This data set consists of 864 images representing 54 classes of natural objects or scenes captured under non-controlled conditions with a variety of devices. From each of the 54 classes, one image has been chosen as representative of the corresponding group. In Experiment 4 we use texture images belonging to the Raw Food Texture database (RawFooT) [48, 49]. It includes images of 68 samples of food textures, acquired under 46 lighting conditions. In our work we have used as stimuli 58 texture images acquired under the D65 lighting condition and frontal direction (TXT2 dataset).

### Participants

Participants were recruited from the Informatics Department of the University of Milano Bicocca and were either students, researchers or administrative employees. No participants under the age of 18 were involved in our study and no health or medical data was collected from participants.

Through the web interface, informed consent was given by all participants. The data was collected anonymously.

For all experiments, six Ishihara tables were preliminarily presented to the observers to estimate color vision deficiency. If the participants did not report correctly any of the six they were discarded from the subjects’ pool.

In Experiment 1, 28 observers participated, and 2 of them failed the Ishihara test. 26 observers remained: 15 women, mean age 37, range 18–53. In Experiment 2, 39 observers different from those of Experiment 1 were involved, and 3 of them failed the Ishihara test. 36 observers remained: 16 women, mean age 33, range 18–53. In Experiment 3, from the 19 initial observers, 17 remained after the control test: 5 women, mean age 35, range 23–51. In Experiment 4, from the 25 initial observers, 23 remained after the control test: 10 women, mean age 28, range 19–68.

All the experiments reported in this article were conducted in accordance with the Declaration of Helsinki and the local guidelines of the University of Milano Bicocca (Italy). No ethical approval was required for the present study. All the stimuli and subjective data are available at our web site [50].

### Experimental setup

In all four experiments observers were asked to judge images individually presented on a web-interface.

**Fig 1:**
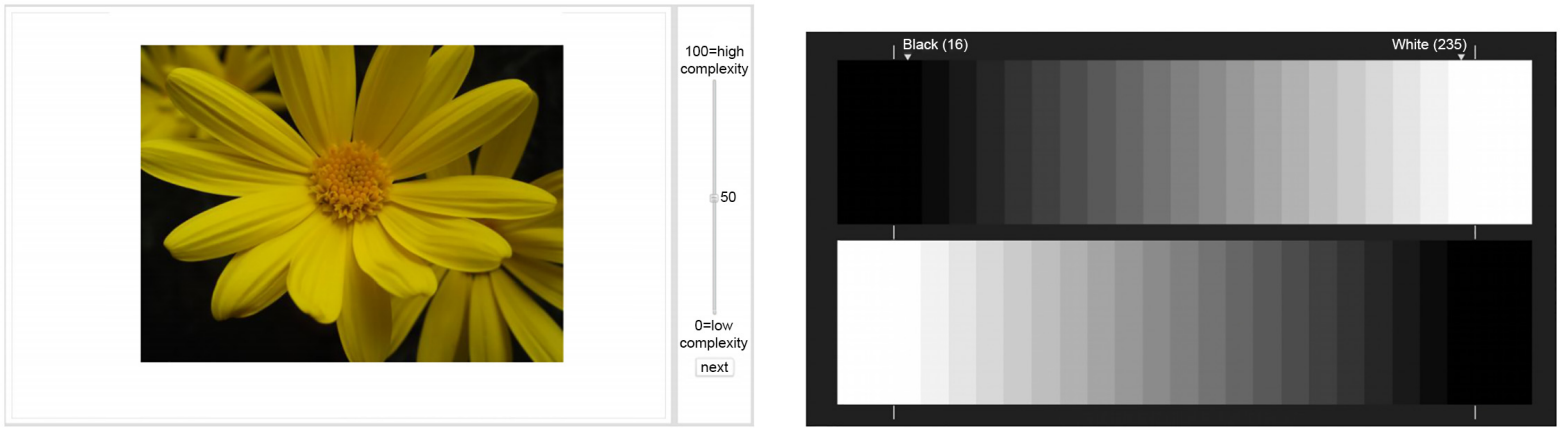
Web-interface of the experiment (left) and contrast chart used to calibrate the monitor (right).

Before the start of the experiment, a grayscale chart was shown to allow the observers to calibrate the brightness and the contrast of the monitor. The observers were asked to regulate the contrast of their monitor to distinguish the maximum number of bands and discern details in shadows and in highlights. In Fig 1 we report the web-interface and the contrast chart used in the experiment.

After calibration, the stimuli were shown in random order, different for each subject. Subjects reported their complexity judgment (score) by dragging a slider onto a continuous scale in the range [0–100]. Stimuli were presented for an unlimited time, up to response submission. The position of the slider was automatically reset after each evaluation at the midpoint of the scale.

In order to get the observers accustomed to the experiment, seven practice trials were presented at the beginning of each experiment, with images not included in the dataset. The corresponding data were discarded and not considered for any further analysis. At the end of the experimental session, the observers were asked to verbally describe the characteristics of the stimuli that affected their evaluation of visual complexity.

### Subject scoring

Mean subjective scores were computed for each observer. The raw, subjective, complexity score *r*_*ij*_ for the *i*-th subject (*i* = 1, …*S*, with *S* = number of subjects) and *j*-th image *I*_*j*_ (*j* = 1, …*N*, with *N* = number of dataset images) was converted into its corresponding Z-score as follows:

$$\begin{array}{c} z_{ij}=\frac{r_{ij}-\bar{r_{i}}}{\sigma_{i}} \end{array}$$ (1)

where $\bar{r_{i}}$ and *σ*_*i*_ are the mean and the standard deviation of the complexity scores over all images ranked by the *i* − *th* subject.

Data were cleaned using a simple outlier detection algorithm. A score for an image was considered to be an outlier if it fell outside an interval of two standard deviations width about the mean score for that image across all subjects.

The remaining Z-scores, were then averaged across subjects to yield the mean scores *y*_*j*_ for each image *j*:

$$\begin{array}{c} y_{j}=\frac{1}{S}\sum_{i=1}^{S}z_{ij} \end{array}$$ (2)

### Our proposal of complexity measure

Due to the multi-dimensional aspect of complexity, we here propose a complexity measure based on a linear combination of *K* different features related to spatial, frequency and color properties. This Linear Combination (*LC*) can be written as follows:

$$\begin{array}{c} LC\left(I_{j}\right)=\sum_{k=1}^{K}a_{k}\times M_{k}\left(I_{j}\right) \end{array}$$ (3)

where *I*_*j*_ is the *j* − *th* image of the considered dataset (*j* = 1, …*N*), and *M*_*k*_ is the measure of the *k* − *th* feature. Let *x*_*j*_ = *LC*(*I*_*j*_) and $x_{kj}=M_{k}\left(I_{j}\right)$, Eq (3) can be rewritten in a compact way as:

$$\begin{array}{c} x_{j}=\sum_{k=1}^{K}a_{k}\times x_{kj} \end{array}$$ (4)

The set of optimal parameters {*a*_*k*_} = *A*^⋆^ ∈ ℜ^*K*^ of Eq (4) were chosen in order to optimally fit subjective data using a population based stochastic optimization technique, called Particle Swarm Optimization (PSO) [38, 39].

In PSO, a population of individuals is initialized as random guesses to the problem solution and a communication structure is also defined, assigning neighbors for each individual to interact with. These individuals are candidate solutions. An iterative process to improve these candidate solutions is set in motion. The particles iteratively evaluate the fitness of the candidate solutions and remember the location where they had their best success. The individual’s best solution is called the particle best. Each particle makes this information available to its neighbors. They are also able to see where their neighbors have had success. Movements through the search space are guided by these successes. The swarm is typically modeled by particles in multidimensional space that have a position and a velocity. These particles fly through hyperspace and have two essential reasoning capabilities: their memory of their own best position and their knowledge of the global or their neighborhood’s best position. Members of a swarm communicate good positions to each other and adjust their own position and velocity based on these good positions.

Recalling that one of the criteria widely used to evaluate the performance of a measure to fit subjective data is the linear Pearson Correlation Coefficient (PCC), we have chosen it as the fitness function to be maximized. To take into account the non linear mapping between objective and subjective data, the complexity measure *x*_*j*_ is previously transformed using a logistic function *f* [51].

The fitness function is thus:

$$\begin{array}{c} PCC\left(A\right)=\frac{\sum_{j=1}^{N}(f\left(x_{j}\right)-\bar{f(x)})\left(y_{j}-\bar{y}\right)}{\sqrt{\sum_{j=1}^{N}{\left(f\left(x_{j}\right)-\bar{f(x)}\right)}^{2}}\sqrt{\sum_{j=1}^{N}{\left(y_{j}-\bar{y}\right)}^{2}}} \end{array}$$ (5)

where *A* is a feasible solution, *f*(*x*_*j*_) is the logistically transformed value of the combined objective measure *LC* for the *j*-th image, and $\bar{f(x)}$ and $\bar{y}$ are the means of the respective data sets.

The optimal parameter values *A*^⋆^ are thus obtained as:

$$\begin{array}{c} A^{⋆}=\max_{A\in {ℜ}^{K}}\left(r(A)\right) \end{array}$$ (6)

Note that our fitness function introduces a simple form of regularization of the searched model that is able to mitigate possible overfitting. In fact, optimizing the PCC defined in Eq (5) we are looking for the solution that minimized the square errors between subjective data and a monotonic curve described by the logistic function.

To benchmark our proposal we consider two clutter measures developed by Rosenholtz et al. [27]. Both of them are defined as a combination of different image features. They were not specifically designed to predict image complexity but have been frequently used in the literature as they show good correlation with complexity perception [3, 32]. The MATLAB implementation provided by the authors has been used:

- Feature Congestion (*FC*): three clutter maps for the image, representing color, texture and orientation congestion are evaluated across scales and properly combined to get a single measure.
- Subband Entropy (*SE*): it is related to the number of bits required for subband image coding. After decomposing the luminance and the chrominance channels into wavelet subbands, the entropy is is computed within each band and a weighted sum of these entropies is proposed as clutter measure.

### Objective measures

In what follows we list and briefly describe the 11 measures used in the linear combination of Eq (3). These measures evaluate simple visual features and were chosen as they were already used as complexity measures in the literature or could be related to complexity. The first six are computed on grayscale images and do not take into account the color information. $M_{1}$ to $M_{4}$ measure properties of the Grey Level Co-occurrence Matrix (GLCM), which is one of the earliest techniques used for image texture analysis and classification [52] and the MATLAB function *graycoprops* was used to compute them.

$M_{1}$:Contrast; it measures the intensity contrast between a pixel and its neighbors over the whole image.
$M_{2}$:Correlation; it measures how correlated a pixel is to its neighbors over the whole image.
$M_{3}$:Energy; it is the sum of squared elements in the GLCM.
$M_{4}$:Homogeneity, it measures the closeness of the distribution of elements in the GLCM with respect to the GLCM diagonal.
$M_{5}$:Frequency Factor, it is the ratio between the frequency corresponding to the 99% of the image energy and the Nyquist frequency [53].
$M_{6}$:Edge Density, it is obtained applying the Canny edge detector to the grayscale image with the parameters indicated by Rosenholtz et al. [27].

The following two $M_{7}$ to $M_{8}$, describe image features which take into account color information when present:

$M_{7}$:Compression Ratio, it is here evaluated as the ratio of the image JPEG compressed with Q factor = 100 and the full size uncompressed image [54].
$M_{8}$:Number of Regions calculated using the mean shift algorithm [55].

Finally, measures from $M_{9}$ to $M_{11}$ evaluate mainly color image properties:

$M_{9}$:Colorfulness; it consists in a linear combination of the mean and standard deviation of the pixel cloud in the color plane [56].
$M_{10}$:Number of Colors; measures the number of distinct color in the RGB image, as described in [57].
$M_{11}$:Color Harmony; based on the perceived harmony of color combinations. It is composed of three parts: the chromatic effect, the luminance effect, and the hue effect [58] [57]:.

## Results

### Experiment 1

**Fig 2:**
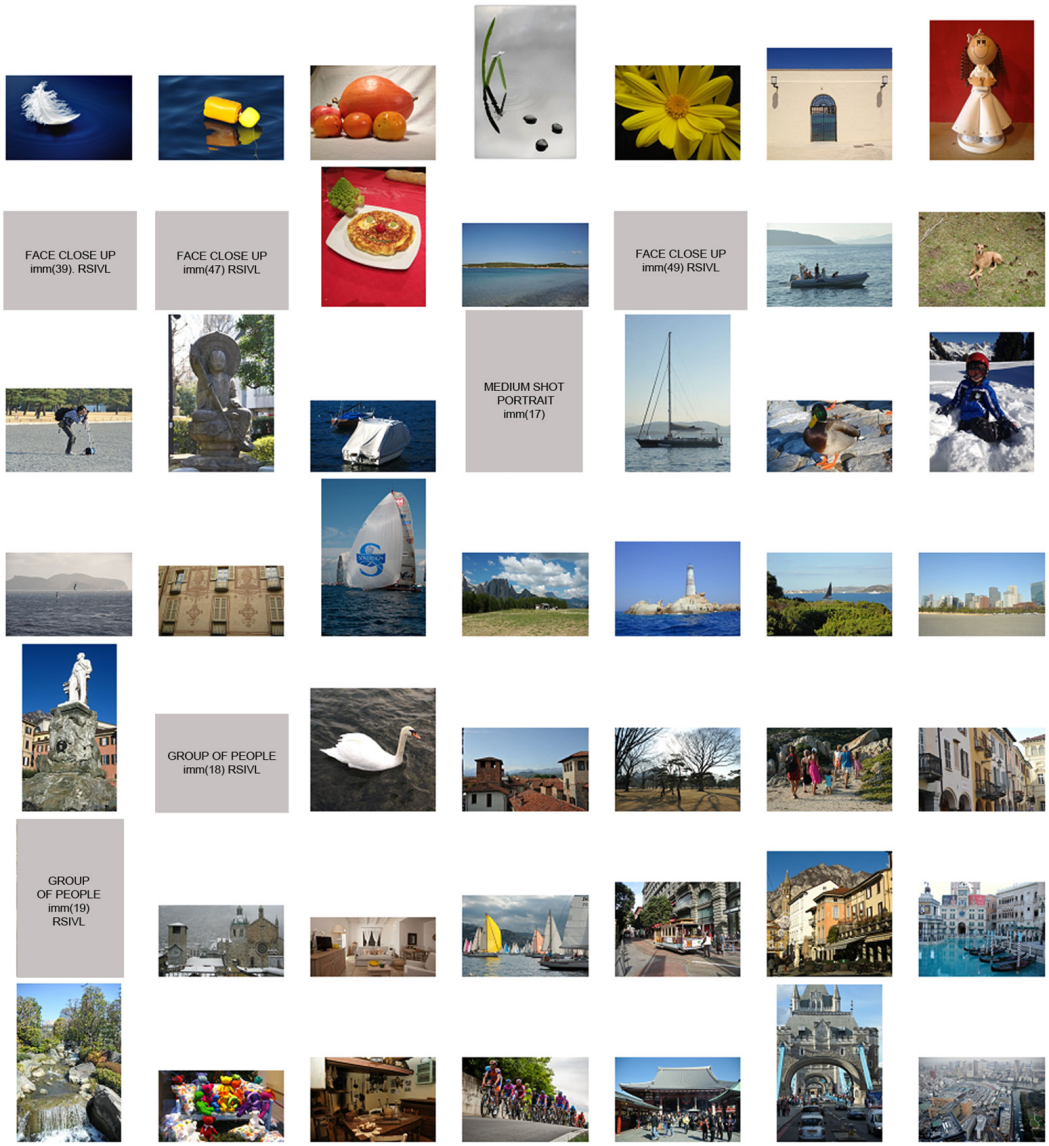
Ranking of the 49 images of the RS1 dataset, according to increasing subjective score. Lowest score = 7, top left, highest score = 89, bottom right. Images with faces or recognizable people are not depicted, but are labeled with the name in the corresponding database.

In Experiment 1 we use the 49 images of real world scenes, belonging to the RS1 dataset. The subjective data collected is processed to obtain the mean scores (see Eq (2). The RS1 images, ordered with respect to increasing mean scores, are reported in Fig 2. Image on the top left corresponds to the minimum mean score, while image on the bottom right is the one with the highest one.

We now use the mean scores of Experiment 1 in the PSO optimization to set the optimal parameters *A*^⋆^ = {*a*_*k*_} of our complexity measure (Eq (3)). To this end, we have performed 1000 runs of the PSO, with normalized measures in the range [0, 1]. The search space of each parameter is in the range [-1 1], to take into account the two possible monotonicities of the 11 single measures combined. Within the 1000 runs, the average PCC (fitness function of PSO optimization, Eq (5)) is 0.85 with standard deviation of 0.012 (minimun PCC = 0.79, maximun PCC = 0.87). These values indicate the convergence of the sequence of solutions. The optimal parameters are thus obtained averaging the 1000 solutions and are reported in Table 1. We call the linear combination obtained using these parameters *LC*_*RS*1_.

**Table 1:** Parameters for Eq (3), obtained averaging the *A*^⋆^ parameters over 1000 runs of PSO.

*a*_1_	*a*_2_	*a*_3_	*a*_4_	*a*_5_	*a*_6_	*a*_7_	*a*_8_	*a*_9_	*a*_10_	*a*_11_
0.2076	0.1986	-0.4046	0.1305	0.5548	-0.1245	-0.1257	0.6167	-0.2588	0.4645	-0.0692

**Fig 3:**
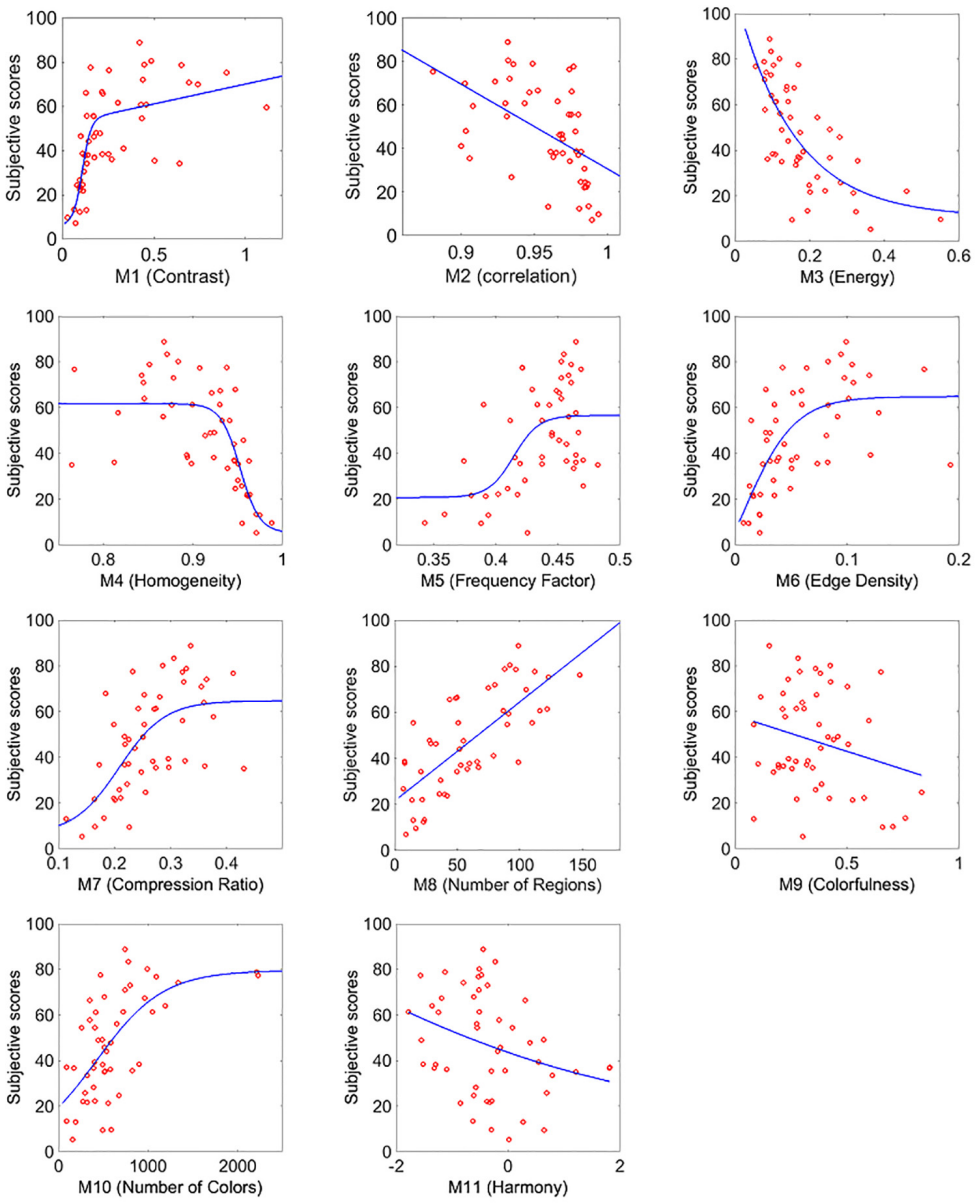
Scatter plots between mean scores and each of the single 11 measures for RS1 dataset.

Since the single measures used to obtain the linear combination *LC*_*RS*1_ have been previously normalized, from Table 1 we can infer the role of each of them when predicting image complexity. The highest contribution to the linear combination comes from $M_{8}$ (Number of regions), followed by $M_{5}$ (Frequency Factor) and $M_{10}$ (Number of Colors) while measure $M_{11}$ (Harmony) is the one with lowest weight. The sign of the coefficients mainly depends on two different aspects. The first one is related to how each single measure correlates with the subjective evaluations. In Fig 3 the scatter plots between mean scores and each of the 11 single measures are shown together with the monotonic functions that best fit the data. Some of the measures show a monotonically increasing correlation while other a monotonically decreasing one. The second aspect is related to the partial correlation between some features. A minus in the linear combination can also take into account the attempt of the PSO algorithm to reduce the redundancy.

**Fig 4:**
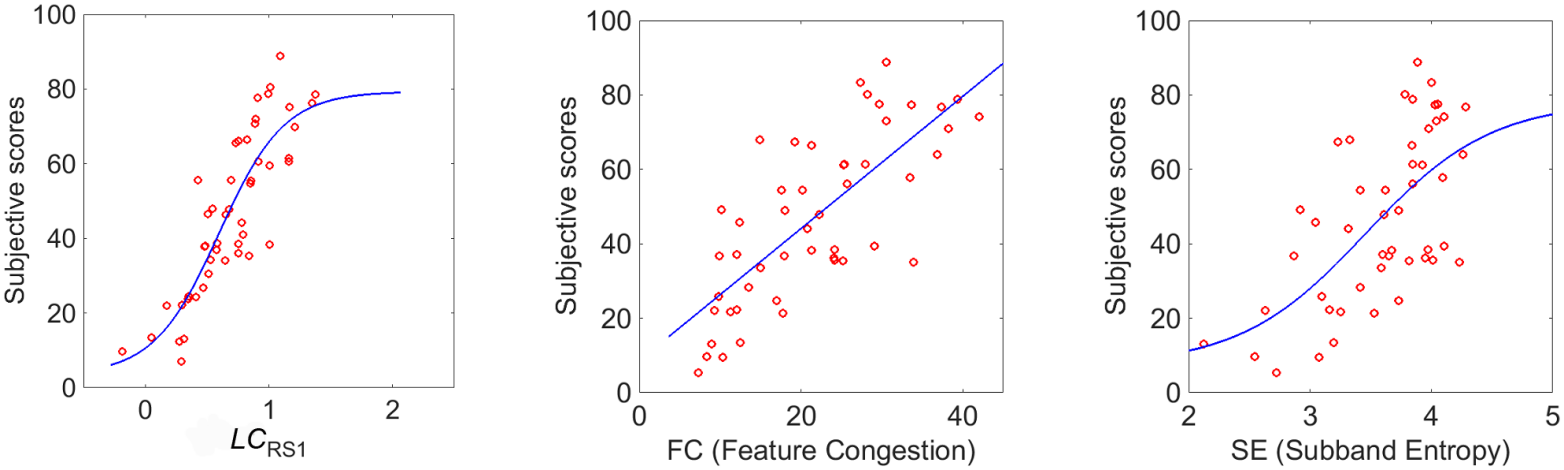
Scatter plots between mean scores and *LC*_*RS*1_, *FC* and *SE* measures.

In Fig 4 the scatter plot between subjective (mean scores) and objective (*LC*_*RS*1_) data of the RS1 images is reported. The monotone function that best fits the data is also shown with a continuous line. To benchmark our proposal, we also plot in the same Figure the scatter plots between mean scores and *FC* and *SE* respectively. To quantify the performance of these complexity measures to correlate with the subjective data, we show in Table 2 (first row) the corresponding PPCs. The p-values are all *p* < 0.001. From the comparison, we observe that *LC*_*RS*1_ outperforms both *FC* and *SE*.

**Table 2:** Correlation performance in terms of PCCs for *LC*_*RS*1_, Feature Congestion and Subband Entropy measures for RS1, RS2, TXT1 and TXT2 datasets.

Measure	*LC*_*RS*1_	Feature Congestion (*FC*)	Subband Entropy (*SE*)
PCC-RS1	0.86	0.75	0.64
PCC-RS2	0.81	0.74	0.66
PCC-TXT1	0.36	0.55	0.44
PCC-TXT2	0.66	0.71	0.15

To better analyze the results, we report in Table 3 (first row) the correlation performance, expressed in terms of PCCs, of each single measure. The p-values are all *p* < 0.001 except for $M_{9}$ and $M_{11}$ where *p* < 0.1. The results show that our proposal outperforms each single metric and confirm our initial hypothesis that a pool of measures can better predict image complexity perception.

**Table 3:** Correlation performance in terms of PCCs for each of the single measures for RS1, RS2, TXT1 and TXT2 datasets.

Measure	$M_{1}$	$M_{2}$	$M_{3}$	$M_{4}$	$M_{5}$	$M_{6}$	$M_{7}$	$M_{8}$	$M_{9}$	$M_{10}$	$M_{11}$
PCC-RS1	0.75	0.52	0.72	0.56	0.57	0.67	0.65	0.74	0.25	0.63	0.30
PCC-RS2	0.64	0.47	0.56	0.64	0.45	0.66	0.67	0.69	0.35	0.43	0.42
PCC-TXT1	0.43	0.19	0.53	0.42	0.35	0.58	0.50	0.47	0.24	0.44	0.14
PCC-TXT2	0.64	0.66	0.77	0.64	0.57	0.47	0.59	0.62	0.14	0.37	0.37

The verbal descriptions recorded during Experiment 1 were mapped into a list of criteria that aggregates concepts with the same meaning. We summarize in Table 4 the most common criteria used, in terms of their frequency with respect to the observers. We underline that each observer could have used more than one criteria. The *quantity of objects, details* and *colors* are the criteria that seem to dominate the complexity perception in Experiment 1. Moreover the most frequent criteria *quantity of objects* of the verbal descriptions is in accordance with the highest coefficient *a*_8_ obtained with the PSO.

**Table 4:** Summary of verbal descriptions corresponding to Experiment 1.

Criterion	Frequency
Quantity of objects	48%
Quantity of details	33%
Quantity of colors	24%
Order and regularity	19%
Understandability	9%

### Experiment 2

**Fig 5:**
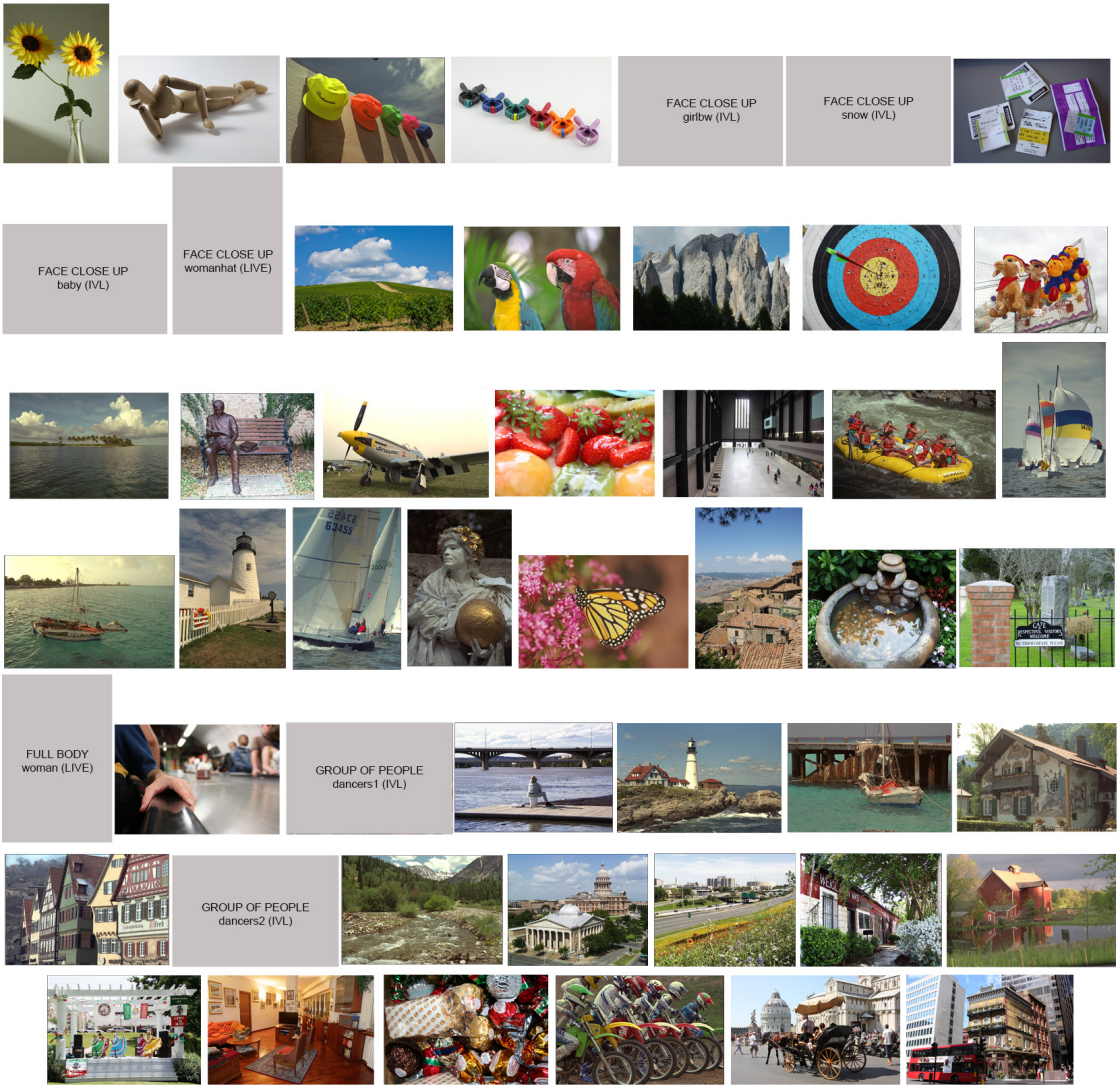
Ranking of the 49 images of the RS2 dataset according to increasing mean scores. Lowest mean score = 19 top left, highest mean score = 74, bottom right. Images with faces or recognizable people are not depicted, but are labeled with the name in the corresponding database.

Experiment 2 is used to test the linear combination *LC*_*RS*1_. The 49 images here considered (RS2 dataset), depict real world scenes. As in Experiment 1, the subjective data is processed to obtain the mean scores. The RS2 images, ordered with respect to increasing mean scores, are reported in Fig 5. Image on the top left corresponds to the minimum mean score, while image on the bottom right is the one with the highest one.

**Fig 6:**
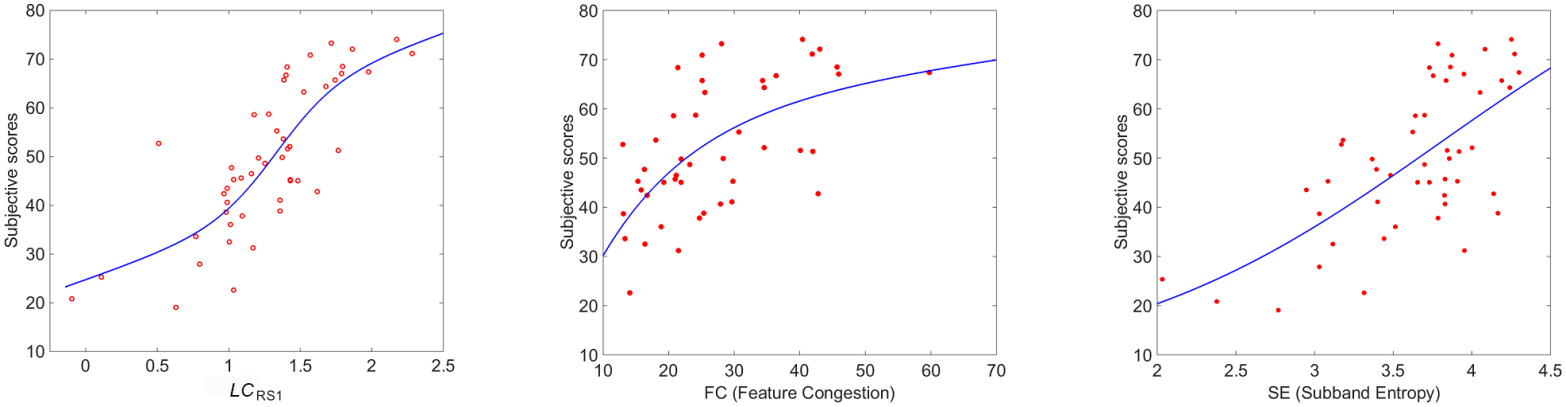
Scatter plots between subjective sscores and *LC*_*RS*1_, *FC* and *SE* measures, respectively on RS2.

The scatter plot between mean scores and our complexity measure (*LC*_*RS*1_) is reported in Fig 6. The monotone function that best fits the data is also shown with a continuous line. The scatter plots between mean scores and *FC* and *SE* respectively are also included in the Figure for a comparison. In Table 2 (second row) the corresponding PPCs are reported. The p-values are all *p* < 0.001. From the comparison, we observe that *LC*_*RS*1_ still outperforms both *FC* and *SE*.

**Fig 7:**
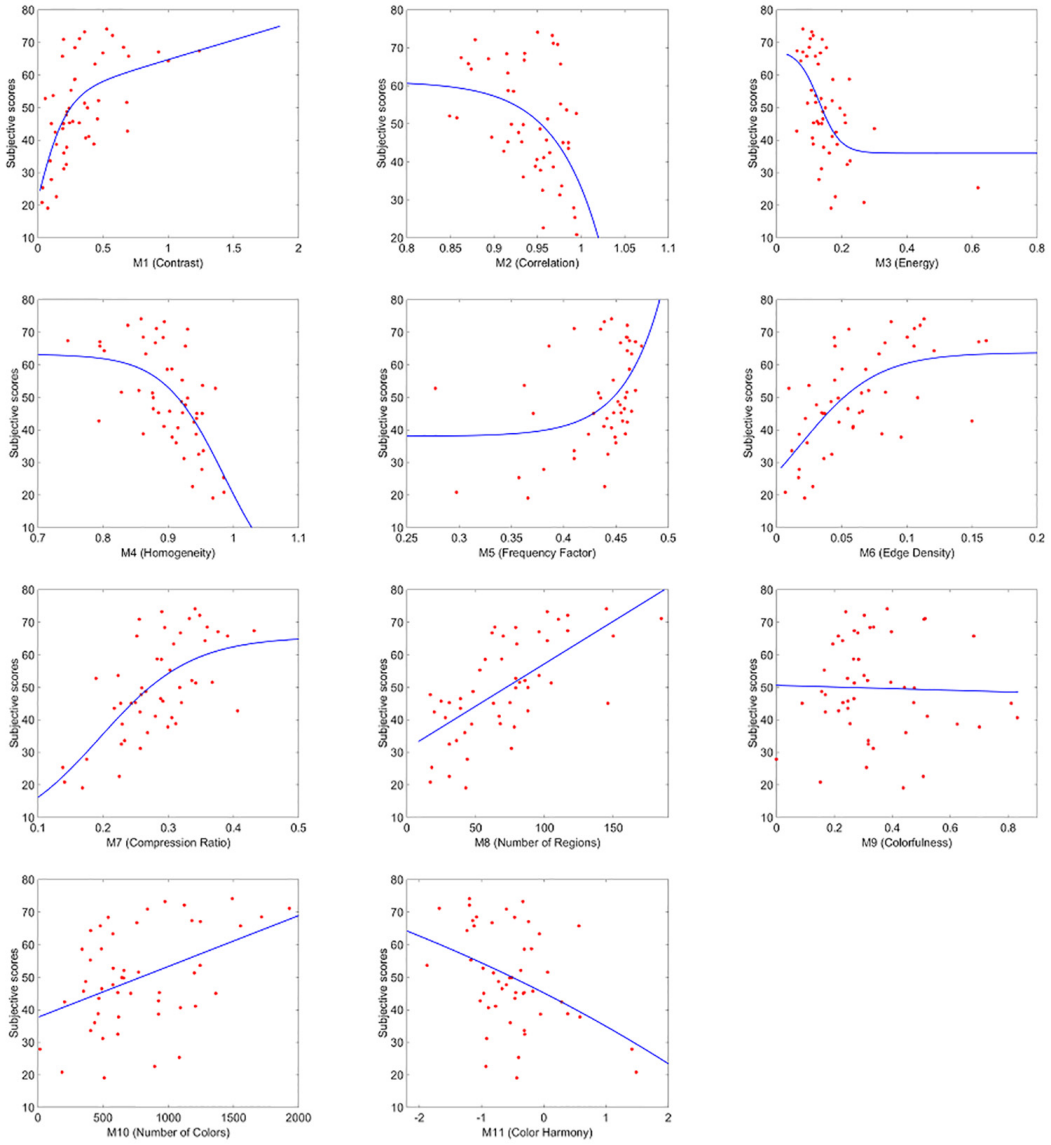
Scatter plots between mean scores and the single measures in the case of RS2 dataset.

In Fig 7 the scatter plots and the best fitting functions between each single measure and the mean scores of Experiment 2 are shown. The correlation performance in terms of PCCs is reported in Table 3 (second row). We observe that in general the performances are decreased with respect to the previous case (RS1 dataset). Measure $M_{8}$ (Number of Regions) confirms to be among those with higher performance, together with $M_{7}$ (Compression Ratio). Measures $M_{9}$ and $M_{11}$, developed mainly to evaluate color properties, show the lowest correlations with p-values *p* < 0.1. For the remaining metrics the p-values are all *p* < 0.001.

Table 5 summarizes the most common criteria collected during Experiment 2. From the analysis of the table we confirm the results of Experiment 1. As in the first Experiment the most relevant criteria used during the complexity assessment are *quantity of objects, details* and *colors*. They are also adopted with similar frequencies by the observers. Two more criteria are also used: *familiarity* and *texture*.

**Table 5:** Summary of verbal descriptions corresponding to Experiment 2.

Criterion	Frequency
Quantity of objects	53%
Quantity of details	31%
Quantity of colors	31%
Understandability	22%
Order and regularity	19%
Familiarity	16%
Texture	12%

### Experiment 3

**Fig 8:**
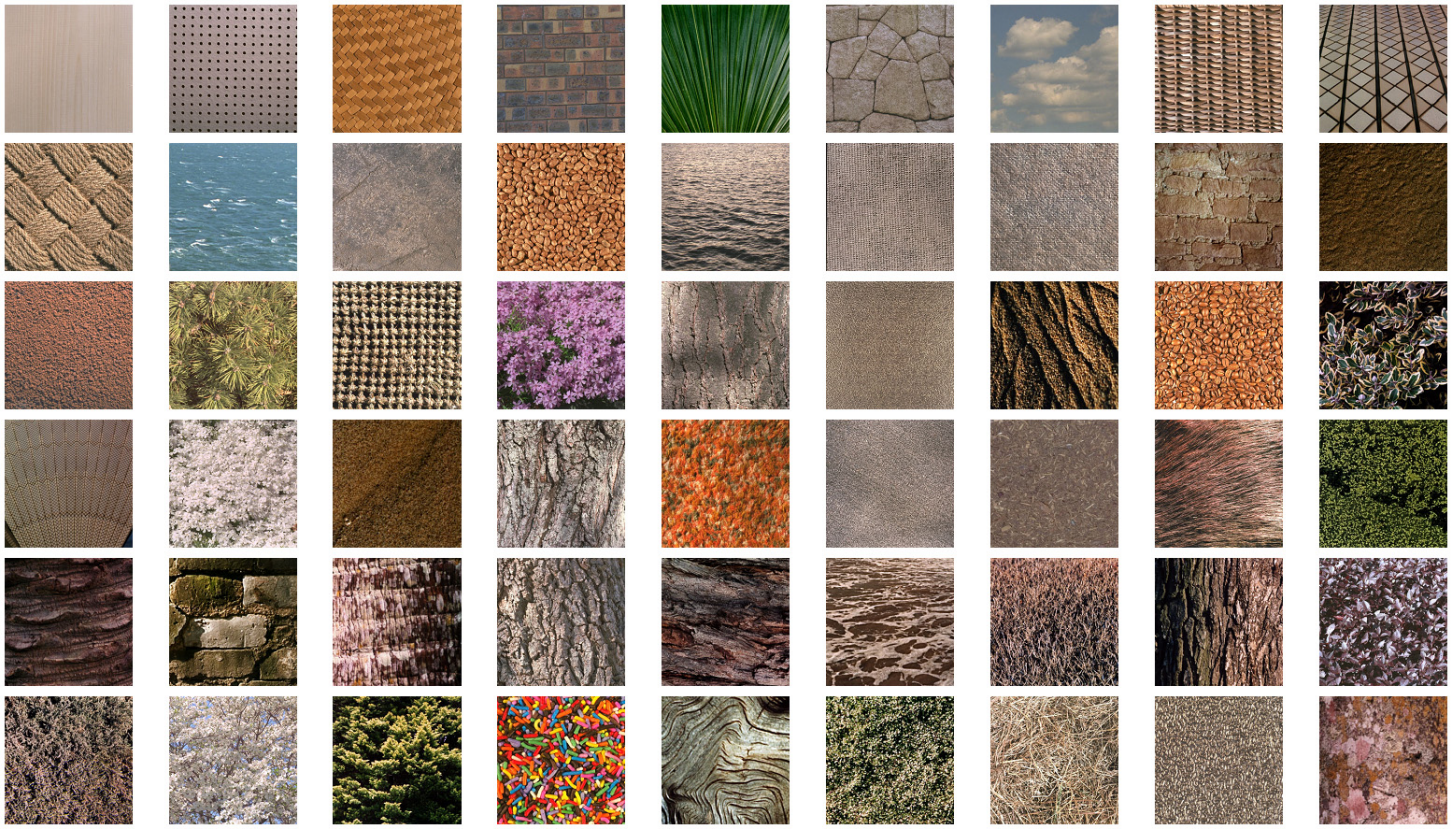
Texture images of TXT1 dataset, ordered from the less complex (top left) to the most complex (bottom right), according to the mean scores.

In Experiment 3 we investigate how the complexity measure *LC*_*RS*1_ behaves when a different kind of stimuli is used. We thus consider as stimuli the 54 texture images of TXT1 dataset. Mean scores are obtained as in Experiments 1 and 2. In Fig 8 stimuli are shown in increasing order of complexity, according to mean scores. Image on the top left corresponds to the minimum mean score, while image on the bottom right has the highest one.

We therefore correlate the linear combination *LC*_*RS*1_ with the subjective data collected for the texture stimuli and we find that it does not perform well: *PCC* = 0.36 with *p* = 0.001. For a comparison, in Table 2 (third row) we report also the PPCs corresponding to *FC* and *SE* applied to the TXT dataset. The p-values are all *p* < 0.001. We observe that also for FC and SE the correlation performance is decreased with respect to the case of real world scenes datasets.

Given the different kind of stimuli here used (texture vs real scenes) we propose to tune the weighting coefficients of the linear combination on this new set of stimuli. As before, we have performed 1000 runs of the PSO to obtain the new set of parameters. Within the 1000 runs, the average PCC results equal to 0.79 with standard deviation equal to 0.02 (minimun PCC = 0.67, maximun PCC = 0.83). In Table 6 we report the 11 weighting coefficients averaged over the 1000 run. We call the linear combination obtained using these coefficients *LC*_*TXT*1_.

**Table 6:** Weighting coefficients for the linear combination *LC*_*TXT*1_, obtained after averaging the parameters over 1000 runs of PSO.

*a*_1_	*a*_2_	*a*_3_	*a*_4_	*a*_5_	*a*_6_	*a*_7_	*a*_8_	*a*_9_	*a*_10_	*a*_11_
0.3653	0.0913	-0.0161	0.0127	-0.7150	0.3961	0.2774	-0.0358	-0.0099	0.4854	0.0209

**Fig 9:**
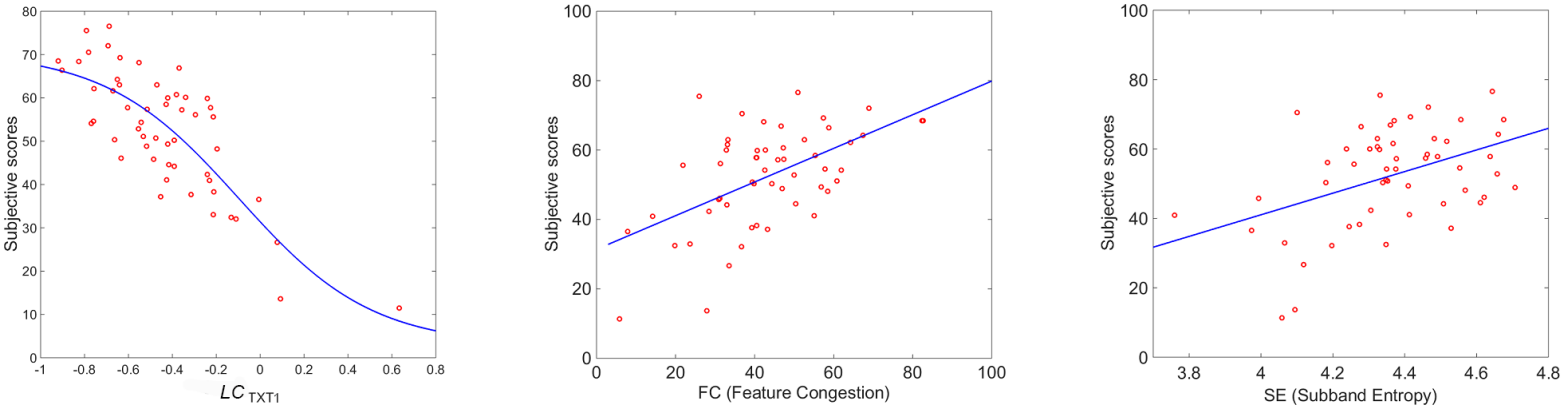
Scatter plots between mean scores of Experiment 3 and *LC*_*TXT*1_, *FC* and *SE* respectively.

Comparing Tables 1 and 6, we observe that the linear combinations *LC*_*RS*1_ and *LC*_*TXT*1_ reflect the different nature of the stimuli (RS versus TXT) in the sign and absolute values of the coefficients. The highest contribution to the linear combination in *LC*_*TXT*1_ comes from $M_{5}$ (Frequency Factor) followed by $M_{10}$ (Number of Colors) and $M_{6}$ (Edge Density). The correlation coefficient PCC between *LC*_*TXT*1_ and the mean scores is now increased and is equal to 0.81. The scatter plots between the mean scores of Experiment 3 and *LC*_*TXT*1_, *FC* and *SE* as well as the best fit of the data are shown in Fig 9 respectively. In Table 7 we summarize the results reporting the PCCs of *LC*_*TXT*1_, *LC*_*RS*1_, *FC*, and *SE* applied to TXT1 dataset. The p-values are all *p* < 0.001. We observe that the linear combination tuned on the texture set of stimuli outperforms all the others.

**Table 7:** Correlation performance in terms of PCCs for *LC*_*TXT*1_, *FC* and *SE* on TXT1 dataset.

Measure	*LC*_*TXT*1_	*LC*_*RS*1_	*FC*	*SE*
PCC	0.81	0.36	0.55	0.44

We also correlate the subjective data collected for the TXT1 dataset with the 11 single measures, reporting in Table 2 (third row) their performance. We observe that in general the single measures do not perform very well. Moreover, for measures $M_{2}$ (Correlation) and $M_{11}$ (Color Harmony) we were not able to find a significant correlation. Only three of them show PCC greater or equal to 0.5 with p-values *p* < 0.001. These three measures are: $M_{3}$ (Energy), $M_{6}$ (Edge Density) and $M_{7}$ (Compression Ratio). For the remaining measures the p-values are *p* < 0.01.

We summarize in Table 8 the verbal descriptions of the observers. With this kind of stimuli the most frequent criteria adopted are regularity, understandability, quantity of details and familiarity, in agreement with the results of Guo et al. [34].

**Table 8:** Summary of verbal descriptions corresponding to Experiment 3.

Criterion	Frequency
Regularity	60%
Understandability	47%
Quantity of details	33%
Familiarity	13%
Quantity of colors	12%

## Experiment 4

**Fig 10:**
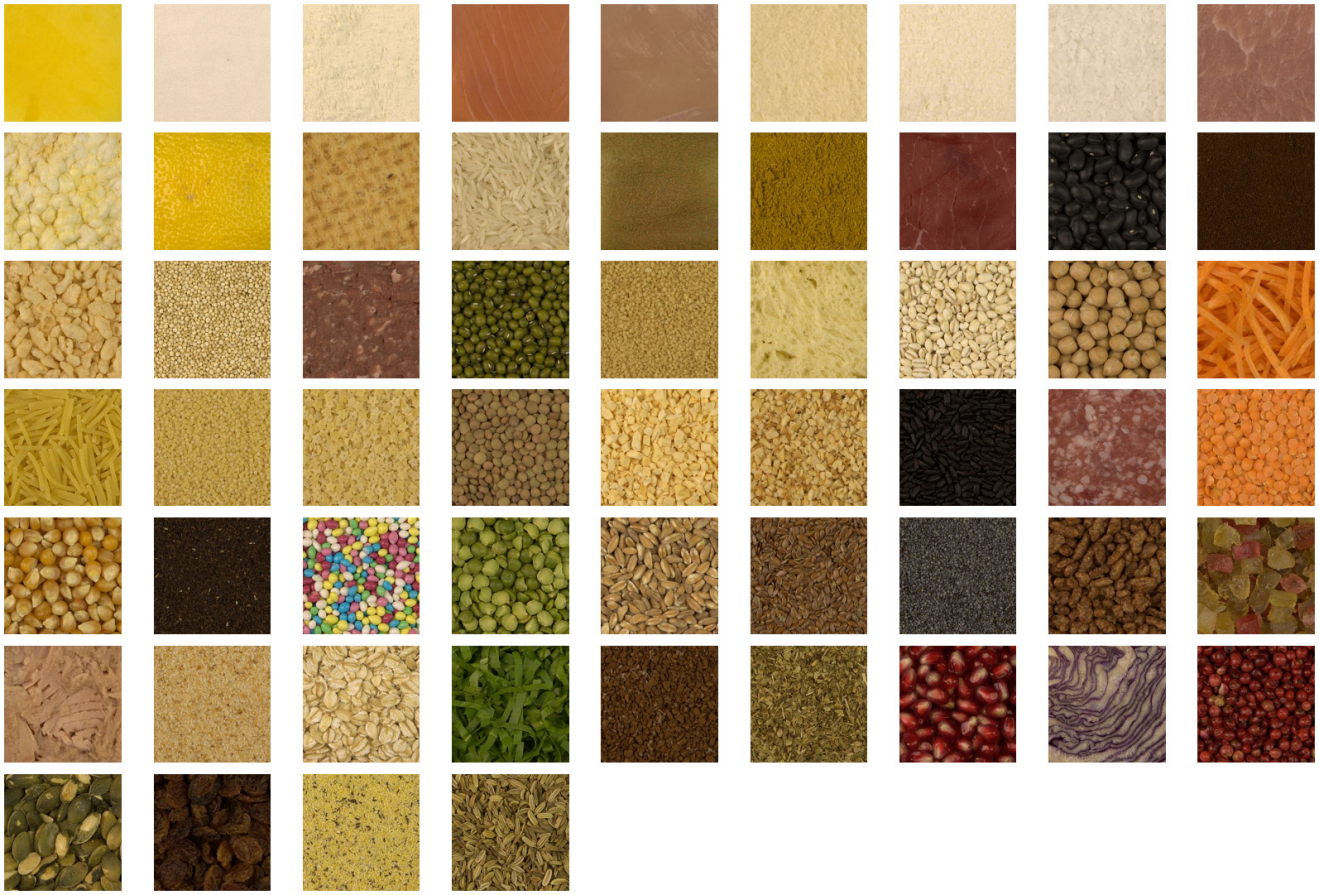
Texture images of TXT2 dataset, ordered from the less complex (top left) to the most complex (bottom right), according to the mean scores.

Experiment 4 is used to further test the linear combination *LC*_*TXT*1_. The 58 images of TXT2 dataset used in Experiment 4 are reported in increasing order of complexity in Fig 10. Subjective scores of this experiment are used to test the linear combination *LC*_*TXT*1_. Its correlation performance is shown in Table 9 and it is compared with the performance on the training set TXT1. Our results confirm that also for the case of texture stimuli, the linear combination proposed *LC*_*TXT*1_ outperforms on the test set all the single measures considered (11 measures, FC and SE) and the *LC*_*RS*1_, as it can be seen from Tables 2 and 3.

**Table 9:** Correlation performance in terms of PCCs for *LC*_*TXT*1_, on TXT1 and TXT2 datasets.

*LC*_*TXT*1_	TXT1	TXT2
PCC	0.81	0.81

## Discussions

From our investigation two aspects of image complexity can be underlined. Many different perceptual properties are involved in image complexity evaluation and their relative influence depends on the type of stimuli. These considerations are supported by both our computational proposal and the analysis of the verbal descriptions.

Analyzing the subjective results of all the four experiments we can try to extract some general considerations about image complexity perception. We separate the following analysis with respect to the different kind of stimuli used. In the case of real world scenes, from Figs 2 and 5 we observe that images with few objects and close-ups are judged as the less complex ones, while on the other hand buildings and streetscapes mainly belong to the most complex ones. These results are in agreement with those obtained by Purchase et al. [31], who addressed image complexity within the field of web interface design.

Analyzing the verbal descriptions reported by the observers while evaluating image complexity (Tables 4 and 5) we note that *quantity of objects, details* and *colors* are the criteria that seem to dominate the complexity perception in both Experiments 1 and 2. For Experiment 2, other two criteria are also used: *familiarity* and *texture*. We observe that many of these verbal descriptions agree with the different definitions found in the literature about image complexity and above reported (section Introduction): Snodgrass et al. [17] refer to the visual complexity as the *amount of details* in an image, Heaps and Hande [18] define complexity as the degree of difficulty in providing a verbal description of an image (*understandability*), Forsythe [20] refers that image complexity should be considered in relation to *familiarity*. We also note that similar criteria have been found in the study by Oliva et al. [30] who use indoor scenes as stimuli. In fact, the authors reported that the criteria corresponding to variety and quantity of objects and colors dominated the representation of complexity, followed by concepts like clutter, symmetry, open space, organization and contrast.

Trying to associate these verbal descriptions with the single objective measures, we can associate the criteria *quantity of objects, details* and *colors* to $M_{8}$ (Number of Regions), $M_{10}$ (Number of Colors), and $M_{6}$ (Edge Density) respectively. The description *order and regularity* can be in correspondence with the visual clutter measures *FC* and *SE*. While *quantity of objects, details and colors* and *order and regularity* can be associated to bottom-up cognitive mechanisms, *understandability* and *familiarity* that play also an important role, are clearly related to top-down processes and none of the considered measures alone is able to capture these concepts. Moreover, several observers have reported both types of criteria (bottom-up and top-down), confirming that bottom-up and top-down mechanisms interfere in perception.

Regarding the texture stimuli, from Figs 8 and 9 we can notice that images with regular pattern and symmetries have been judged as less complex, while images with more details and less ordered structures have been judged as more complex. These findings are in accordance with those obtained by Heaps and Handel [36]. They ranked the complexity of 24 texture images, printed in grayscale and belonging to the same VisTex database as ours and they observed that “textures with repetitive and uniform oriented patterns were judged less complex than disorganized patterns”.

The order of importance of the verbal descriptions of Experiment 3 has changed with respect to the corresponding ones of Experiments 1 and 2 (see Tables 4, 5 and 8. For texture images *regularity* is the most relevant criteria (reported by 60% of the observers), followed by *understandability* (47%). Instead for real world scenes these two criteria are among the ones less used (19% and 9% respectively in Experiment 1, and 19% and 22% in Experiment 2). These results are in accordance with those obtained by Yin et al. [25], who used sample images from Brodatz’s album [59]. They found that regularity, understandability, roughness, directionality, and density are the main characteristics that affect the visual complexity perception of texture images.

The differences between complexity perception of real scenes and texture patches are mainly related to the different image content and are reflected by the different order of importance and frequency of the criteria reported in the verbal descriptions. In particular real scene images are more easily understandable than texture images. Analyzing the verbal descriptions reported by the observers we note that understandability is present for all the experiments. However in case of texture images it was more frequent, denoting that probably observers pay more attention on this aspect while evaluating texture complexity. Instead, in case of real world scenes understandability has been used with less frequency, as probably real scenes are intrinsically more understandable.

Comparing the performance of the single features, in terms of PCCs (see Table 3), we observe that in general those obtained for real world datasets (RS1 and RS2) are higher than the corresponding ones obtained for texture images. Following a similar trend, our linear combination proposal trained on real world scenes (*LC*_*RS*1_) shows a significantly low performance applied to texture images (see Table 2). However, we have demonstrated that if the parameters of the linear combination are optimized with respect to this new dataset (TXT1), a significant improvement is reached. The final performance is thus comparable with that previously obtained on real world images.

Also Chikhman et al. [29] concluded that for different types of images, different measures of complexity may be required. In fact they found that for outline objects the best predictor of their experimental data was number of turns of an image, while for the hieroglyphs set of stimuli, the best correlation was given by the product of the square spatial frequency median and the image area. Focusing on streetscape images, Cavalcante et al. [32] proposed to combine contrast and spatial frequency to form a single objective measure. Within the same kind of streetscape images they found that their proposal is more effective and robust for nighttime scenes than for daytime ones and thus they concluded that “objective measures based on reduced sets of low-level image characteristics are unlikely to be satisfactory for all possible streetscapes”.

To assess complexity of painting images, Guo et al. [37] proposed to combine several visual features in a machine learning approach to classify images into three groups of high, medium and low complexity. In this way the authors were able to achieve a high classification performance. Moreover, studying the role of the single feature, they found that features related to hue and local color variations mostly conditioned the classification results in the specific case of painting images.

In our study we have considered real world images that cover several contents, including textures. We have considered eleven visual features, but our computational proposal can be extended either using different features or increasing the number of them, to cope with other different kind of stimuli.

Finally we remark that in our work we have considered databases of high quality images. An interesting aspect that should be investigated in the future is the interference between image quality and complexity perception: how do different kind of distortions influence image complexity perception? How does image complexity influence quality perception? The results of this analysis in fact could give an important insight in interpreting how signals and artifacts mutually interfere while evaluating image complexity.

## Conclusions

Real world images show a high variability in depicted content. When observer are asked to assess image complexity, it emerges that complexity perception is guided by different criteria. Some of them are related to visual features representing bottom-up processes, while others are more related to top-down ones. Moreover, several criteria are adopted simultaneously by each observer, showing a multidimensional aspect of complexity. Thus in this work we have proposed a complexity measure that linearly combines several visual features. When this measure is applied to real world scenes and texture it is able to predict the complexity perception outperforming each single feature. The weighting coefficients of the linear combination depend on the kind of stimuli and the relative contribution of the measures mostly reflects the criteria used by the observers. As a future work, to define a more general complexity measure, we are planning to mix images belonging to real scene and texture databases on which conduct new psycho-physical experiments. Furthermore, we plan to investigate non-linear models to combine the single measures, as for example Support Vector Machine or Genetic Programming to take into account different modalities of interactions of visual properties.

## Supporting Information

S1 File Objective measures and subjective scores of images in the RS1 real world scenes database. (XLS)

S2 File Objective measures and subjective scores of images in the RS2 real world scenes database. (XLS)

S3 File Objective measures and subjective scores of images in the TXT1 texture images database. (XLS)

S4 File Objective measures and subjective scores of images in the TXT2 texture images database. (XLS)
